# Personalized video-based health education after ischemic stroke: A single-center cross-sectional study in China

**DOI:** 10.1371/journal.pone.0316062

**Published:** 2025-11-20

**Authors:** Jing-Xiu Gao, Mei-Ru Wu, Yi-Tong Chen, Yong-Mei Deng, Chun-Juan Wang, Rui-Hua Ma

**Affiliations:** 1 Vascular Neurology, Department of Neurology, Beijing Tiantan Hospital, Capital Medical University, Beijing, China; 2 Nursing Department, Beijing Tiantan Hospital, Capital Medical University, Beijing, China; 3 China National Clinical Research Center for Neurological Diseases, Beijing, China; 4 Research Unit of Artificial Intelligence in Cerebrovascular Disease, Chinese Academy of Medical Sciences, Beijing, China; UCSF: University of California San Francisco, UNITED STATES OF AMERICA

## Abstract

**Background and objectives:**

Health education can help patients engage in evidence-based healthy behaviors, and the construction of smart hospitals can promote accurate video-based health education for patients. However, little is known about the clinical practice of personalized video-based health education for ischemic stroke patients in China. We investigated video-based health education viewing frequency and relevant impact factors among patients with ischemic stroke.

**Methods:**

Data were collected from 1,569 ischemic stroke patients admitted to the Vascular Neurology Ward of a hospital in China between 01/08/2020 and 31/12/2022, to analyze their use of personalized video-based health education. The video-based integrated management system used in our hospital can facilitate keyword extraction, thus achieving accurate and personalized video-based health education and promotion. Data, including demographic and disease-related data, viewing frequency, total viewing amount and viewing frequency for each dimension, were extracted from the hospital’s video integrated management system and medical system. Univariate analysis and multiple linear regression helped identify factors associated with whether the patients viewed personalized video-based health education materials.

**Results:**

A total of 1569 patients were included (age = 58.72 ± 13.50 years old; 1177 (75.0%) males). Diet rehabilitation education was the most frequently viewed personalized video-based health education dimension; the average viewing frequency was 2.30 ± 1.15 times/day during an average hospitalization of 12.55 ± 4.12 days. According to the multivariable analysis, factors associated with a reduced likelihood of viewing the personalized video-based health education materials (P < 0.05) included visual and hearing impairment, longer hospital stays, and speech impairment. In contrast, compared to self-paying patients, individuals who were covered by medical insurance or received free medical service were more likely to view the personalized video-based health education materials.

**Conclusion:**

A personalized video-based health education program with a keyword extraction function can help achieve accurate and personalized video-based health education and promotion and effectively improve patients’ health-education participation rate.

## 1. Introduction

Stroke is a major global health problem and is the second leading cause of death worldwide [1], affecting an estimated 93.2 to 110.5 million people worldwide [2]. Ischemic stroke accounts for approximately 80% of all strokes, and its annual recurrence rate is as high as 9.6% ~ 12.5% [3,4]. To reduce the risk of recurrent stroke, a series of clinical trials have been conducted in routine clinical practice settings to investigate the secondary prevention of vascular events, and the effectiveness of several measures, including the use of antiplatelets, anticoagulants, antihypertensives, hypoglycemics, and statins [5], were demonstrated. Effective secondary prevention strategies are an important means to reduce stroke recurrence, disability and death [6,7]. Effective health education during hospitalization can help patients establish evidence-based healthy behaviors and improve disease prognosis [8]. In the context of the vigorous construction of smart hospitals and the continuous innovation of smart services, many hospitals are equipped with video equipment, and studies have reported that video-based health education can improve the mastery of health knowledge and compliance of stroke patients and improve their prognosis and quality of life [9–11]. In the context of “smart hospitals”, how to incorporate personalized video-based health education and establish links between hospital information systems to accurately promote health education for patients are issues that multidisciplinary teams need to consider [12,13].

To date, research on stroke health education has mainly focused on improving knowledge mastery. However, the results of different studies have varied. For example, Wei et al. reported that hypertension history and education level are influential factors for the health education of patients with stroke [14]. However, Zhong et al. noted that strengthening stroke-related health promotion and incorporating educational activities may increase awareness regarding the need to call emergency services after the onset of stroke signs [15]. In a study of Taiwanese stroke patients, Tang et al. reported that in outpatient clinical practice, nurses can help patients increase their awareness of stroke risk through health education CD-ROMs [16].

At present, there are no relevant studies analyzing the factors influencing the use of video-based health education. We used data from the unified medical video integrated management system and medical records system of a hospital in China. The purpose of this study was to investigate the video-based health education viewing frequency in patients with ischemic stroke, explore the factors influencing the use of personalized video-based health education, and provide a basis for targeted health education and health promotion interventions.

## 2. Methods

### 2.1. Design

This was a single-center cross-sectional study design.

### 2.2. Participants

Patients with ischemic stroke in the vascular neurology ward of a tertiary hospital in China from 01/08/2020 to 31/12/2022 were selected as the study subjects. Patients who met the following criteria were recruited consecutively in a tertiary hospital in China from 01/08/2020 to 31/12/2022: (1) aged 18 years and older; (2) initial diagnosis of ischemic stroke confirmed by cerebral CT or MRI; (3) first stroke; (4) clear consciousness and stable vital signs; and (5) willingness to voluntarily participate and sign an informed consent form. Patients with cognitive dysfunction, mental symptoms, severe aphasia, or vital organ dysfunction were excluded. A total of 1569 patients with ischemic stroke who met the inclusion criteria of this study were enrolled in the final study sample.

### 2.3 Ethical approval and informed consent

This research was conducted in accordance with the guidelines of the Declaration of Helsinki and was approved by the Institutional Review Board (IRB) of Capital Medical University, Beijing Tiantan Hospital, China (Ethical Approval Number: KY2020-016-02). All participants voluntarily participated in this research and signed an informed consent form in person. All methods complied with the relevant guidelines and regulations.

### 2.4. Personalized video-based health education design

#### 2.4.1. Health education path and content design.

Literature [17–19] was retrieved and combined with information on the diagnosis and treatment path of ischemic stroke, the education process for ischemic stroke patients starting from the day of admission, the first day of admission, and the second day of admission to the day of discharge, including information on the environment, intravenous therapy introduction, safety notification, disease education, auxiliary examination, medication guidance, diet education, rehabilitation guidance, perioperative education of neurological intervention, discharge education and other education content. The content related to the mission and education process was formed into health education videos, and medical and nursing teams were organized to review the health education content, and ensure the scientific, accurate and instructive content of the health education materials [20].

#### 2.4.2. Personalized health education program design.

**Fig 1:**
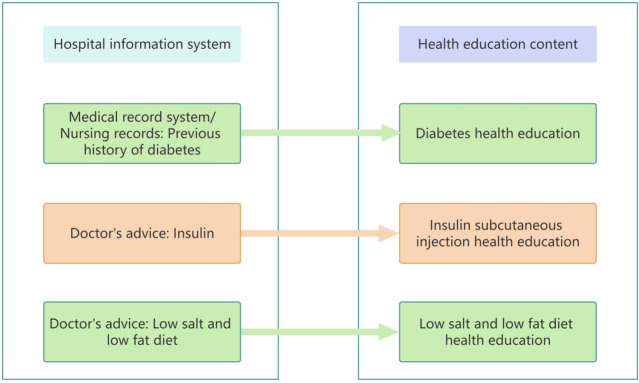
Matching scheme of "Information system keywords-health education content".

A multidisciplinary team of medical treatment, nursing and education professional was established. The medical and nursing team discussed the keywords of the health education content corresponding to the medical records system of the hospital, the order processing system for the doctors, and the nursing records system. The researchers and the information team formulated the keyword extraction scheme and the matching scheme of “keyword - health education content”. Then, combined with the established health education path, the multidisciplinary team discussed the development of a personalized health education bedside digital TV promotion plan (Fig 1).

#### 2.4.3. Implementation *of* personalized video-based health education.

The personalized video-based health education content was divided into 5 dimensions, including discharge health education, admission health education, disease-related medication education, auxiliary examination surgery education, and diet rehabilitation education, for a total of 185 educational videos. The form of health education used in this study was mainly video playback with assistance from a nurse. The bedside digital TV system provided personalized updates to the patients’ health education videos every day. The attending nurse opened the health education videos for the patients every morning, explained the daily health education learning objectives, and assisted the patients in recalling the health education content after watching. After each video-based health education session, there were three simple multiple-choice questions for patients to answer to evaluate the learning effect of the session; whenever a question was answered correctly, a fireworks GIF appeared to encourage the patient.

### 2.5. Outcomes and data collection

The personalized video-based health education viewing data of ischemic stroke patients, including the viewing frequency, the total number of videos viewed and the number of videos viewed in each dimension were extracted from the unified medical video integrated management system of Tiantan Hospital from August 1, 2020 to December 31, 2022. The per capita viewing frequency was defined as follows: total number of videos watched per month/(number of hospitalized patients per month*average length of stay per month). General patient data, including sex, age, nationality, marital status, education level, occupation, type of medical insurance, family monthly income, residence status, previous medical history, family history of stroke, thrombolytic history, smoking history, drinking history, BMI, MMSE score, muscle strength, dysfunction, mRS score within 2 hours of admission, length of stay, and discharge destination, were extracted from the medical records system.

### 2.6. Ethical considerations

The study was approved by the ethics committees of the hospital (KY2020-016-02). Each patient was informed of the purpose, content and procedure of the study when invited to participate. Only those who provided informed consent were included in the study. All patients’ personal information was confidential.

### 2.7. Data analysis

SPSS software version 25.0 (IBM Corp., Armonk, NY, USA) was used for the statistical analysis. *p* values < .05 were considered to indicate statistical significance. T tests and one-way ANOVA were used to assess the differences in the viewing frequency of personalized video-based health education materials between individuals with different demographic and disease characteristics. Least significant difference (LSD) tests were used for pairwise comparisons among three or more groups. Multiple linear regression was used to analyze the related factors significantly influencing the viewing frequency of personalized video-based health education. Factors with *p* values < .10 in the univariate analysis were selected as independent variables, and the viewing frequency of personalized video-based health education materials was used as the dependent variable. Before a classification variable was entered into the multivariable model, it was first converted to a dummy variable.

## 3. Results

### 3.1. Patient characteristics

A total of 1569 patients with ischemic stroke were included in this study, and there were no deaths during the study period. The general characteristics of the study population are described in Table 1.

**Table 1:** Description of the study population (n = 1569).

Variables	n(%)/mean±SD/ median(IQR)	Variables	n(%)/mean±SD/ median(IQR)
**Sex**		**Medical history**	
Male	1177(75.0)	Hypertension	1031(65.7)
Female	392(25.0)	Diabetes	547(34.9)
**Age (years)**	58.72 ± 13.50	CHD	247(15.7)
**Race**		Smoking	929(59.2)
Han	1501(95.7)	Alcoholism	846(53.9)
Non-Han	68(43.3)	Intravenous thrombolysis	215(13.7)
**Marital status**			
Single	28(1.8)	Family history of stroke	439(28.0)
Married	1491(95.0)		
Divorced	23(1.5)	**BMI**	25.40 ± 3.42
Widowed	27(1.7)	**MMSE**	30(28,30)
**Education level**		**Myodynamia**	
Primary and below	421(26.8)	Level 5	732(46.7)
High school	928(59.1)	Level 5- to 3	535(34.1)
College or above	220(14.1)	Below level 3	302(19.2)
**Occupation**		**Dysfunction**	
Worker	124(7.9)	Limb motor dysfunction	1116(71.1)
Peasant	174(11.1)		
Professionals	105(6.7)	Aphasia	653(41.6)
Business service personnel	142(9.1)	Dysphagia	211(13.4)
Administrator	55(3.5)	Voiding dysfunction	53(3.4)
Retirement	887(56.5)	Visual and auditory impairment	288(18.4)
Student	9(0.6)		
Other	73(4.6)	**mRS Score within 2 hours of admission**	
**Insurance status**			
Self-pay	297(18.9)	0-1	1025(65.3)
Medical insurance	1240(79.0)	2-5	544(34.7)
Free medical service	32(2.1)	**Hospitalization length**	12.55 ± 4.12
**Monthly household income**		**Disposition**	
		Discharge to home	1176(75.0)
Less than 5,000 yuan	480(30.6)	Discharge to rehabilitation center	302(19.2)
Five to ten thousand yuan	924(58.9)		
More than 10,000 yuan	165(10.5)	Discharge to level II and below hospitals	91(5.8)
**Living situation**			
Live alone	755(48.1)		
Live with spouse	622(39.6)		
Live with children	183(11.7)		
Live with parents	9(0.6)		

### 3.2. Personalized video-based health education viewing situation

**Fig 2:**
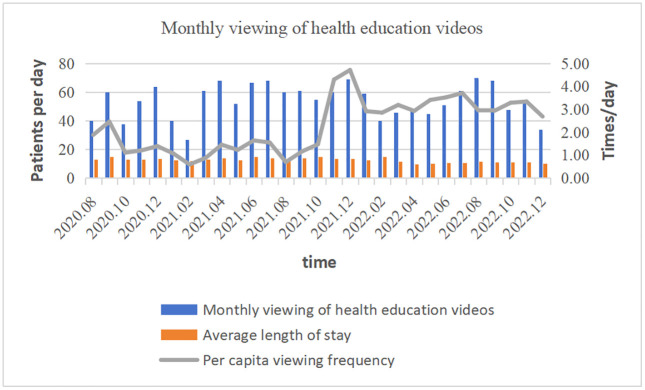
Monthly viewing of health education videos.

From August 1, 2020, to December 31, 2022, the total number of views on 5 dimensions of 185 video-based health education items was 45,255, and the dimension with the most views was diet and rehabilitation education, with 17,602 views. The per capita viewing frequency increased over time, reaching a maximum of 4.72 times/day and an average of 2.30 ± 1.15 times/day (Table 2 and Fig 2).

**Table 2:** Viewing of health education videos.

Item	Number of videos	Number of views
Discharge health education	6	2110
Integrated admission education	24	3574
Disease-related drug education	44	10155
Adjuvant surgery education	49	11814
Diet rehabilitation education	62	17602
Total	185	45255

### 3.3. Predictors of personalized video-based health education viewing

The univariate analysis results are presented in Table 3. Univariate analysis of the clinical data of the patients revealed statistically significant differences in education level, type of medical insurance, cognitive level, muscle strength, dysfunction, mRS score within 2 hours after admission, length of stay and discharge destination on their health education video viewing (*P* < 0.05).

**Table 3:** Results of univariate analysis of the viewing frequency of personalized video-based health education materials among patients with ischemic stroke (N = 1569).

Variables		n (%)	Viewing frequency of personalized video-based health education (times/day), *Mean ± SD*	*t/F* ^a^	*P* ^*b*^
**Sex**				0.866	0.421
Male		1177(75.0)	2.61 ± 1.12		
Female		392(25.0)	2.62 ± 1.56		
**Age**				0.325	0.722
18-45y		185(11.8)	2.62 ± 1.01		
46-60y		567(36.1)	2.69 ± 1.06		
> 60y		817(52.1)	2.58 ± 1.19		
**Race**				0.686	0.602
Han		1501(95.7)	2.61 ± 1.13		
Non-Han		68(43.3)	2.77 ± 1.21		
**Marital status**				1.189	0.314
Single		28(1.8)	2.76 ± 1.22		
Married		1491(95.0)	2.61 ± 1.13		
Divorced		23(1.5)	2.04 ± 1.11		
Widowed		27(1.7)	3.30 ± 0.84		
**Education level**				5.940	0.003^**^
Primary and below①		421(26.8)	2.32 ± 1.18		<②^**^
High school②		928(59.1)	2.78 ± 1.08		<③
College or above③		220(14.1)	2.51 ± 1.10		
**Occupation**				0.951	0.467
Worker		124(7.9)	2.92 ± 1.12		
Peasant		174(11.1)	2.43 ± 1.14		
Professionals		105(6.7)	2.54 ± 1.07		
Business service personnel		142(9.1)	2.28 ± 1.05		
Administrator		55(3.5)	2.56 ± 1.08		
Retirement		887(56.5)	2.68 ± 1.15		
Student		9(0.6)	2.30 ± 0.91		
Other		73(4.6)	2.70 ± 1.11		
**Insurance status**				3.405	0.034^*^
Self-pay①		297(18.9)	2.30 ± 1.10		<②^*^
Medical insurance^c^②		1240(79.0)	2.70 ± 1.13		<③
Free medical service③		32(2.1)	2.66 ± 1.00		
**Monthly household income**				2.112	0.123
Less than 5,000 yuan		480(30.6)	2.68 ± 1.18		
Five to ten thousand yuan		924(58.9)	2.66 ± 1.11		
More than 10,000 yuan		165(10.5)	2.26 ± 1.03		
**Living situation**				1.352	0.258
Live alone		755(48.1)	2.69 ± 1.18		
Live with spouse		622(39.6)	2.61 ± 1.06		
Live with children		183(11.7)	2.31 ± 1.12		
Live with parents		9(0.6)	3.13 ± 1.61		
**Medical history**					
Hypertension	Yes	1031(65.7)	2.64 ± 1.13	0.327	0.568
	No	538(34.3)	2.57 ± 1.12		
Diabetes	Yes	547(34.9)	2.57 ± 1.15	0.517	0.473
	No	1022(65.1)	2.66 ± 1.11		
CHD	Yes	247(15.7)	2.31 ± 1.11	2.860	0.059
	No	1322(84.3)	2.68 ± 1.12		
Smoking	Yes	929(59.2)	2.60 ± 1.10	0.110	0.740
	No	640(40.8)	2.64 ± 1.17		
Alcoholism	Yes	846(53.9)	2.59 ± 1.19	0.348	0.556
	No	723(46.1)	2.66 ± 1.17		
**Intravenous thrombolysis**					
Yes		215(13.7)	2.82 ± 1.10	1.678	0.196
No		1354(86.3)	2.59 ± 1.13		
**Family history of stroke**					
Yes		439(28.0)	2.59 ± 1.15	0.104	0.748
No		1130(72.0)	2.63 ± 1.12		
**BMI**				0.164	0.920
< 18.5		32(2.0)	2.69 ± 1.22		
18.5-23.9		439(28.0)	2.65 ± 1.09		
24-27.9		823(52.5)	2.58 ± 1.13		
≥ 28		275(17.5)	2.69 ± 1.19		
**MMSE**				10.904	0.001^**^
27-30		1340(85.4)	2.70 ± 1.12		
21-26		229(14.6)	2.14 ± 1.07		
**Muscle strength**				9.545	<0.001^**^
Level 5①		732(46.7)	2.89 ± 1.11		>②^**^
Levels 5–3②		535(34.1)	2.45 ± 1.14		>③^**^
Below level 3③		302(19.2)	2.27 ± 1.01		
**Dysfunction**					
Limb motor dysfunction	Yes	1116(71.1)	2.40 ± 1.12	8.419	<0.001^**^
	No	453(28.9)	2.88 ± 1.08		
Aphasia	Yes	653(41.6)	2.23 ± 1.09	35.343	<0.001
	No	916(58.4)	2.92 ± 1.06		
Dysphagia	Yes	211(13.4)	2.08 ± 1.08	15.492	<0.001
	No	1358(86.6)	2.72 ± 1.11		
Voiding dysfunction	Yes	53(3.4)	1.63 ± 0.95	18.132	<0.001
	No	1516(96.6)	2.68 ± 1.11		
Visual and auditory impairment	Yes	288(18.4)	1.45 ± 0.73	109.504	<0.001
	No	1281(81.6)	2.88 ± 1.03		
**MRS Score within 2 hours of admission**				29.507	<0.001^**^
0-1		1025(65.3)	2.85 ± 1.11		
2-5		544(34.7)	2.18 ± 1.03		
**Hospitalization length**				87.236	<0.001^**^
0-12d		997(63.5)	3.00 ± 1.08		
≥13d		572(36.5)	1.95 ± 0.86		
**Disposition**				8.981	<0.001^**^
Discharge to home①		1176(75.0)	2.77 ± 1.10		① > ②^**^
Discharge to rehabilitation center②		302(19.2)	2.19 ± 1.04		① > ③^*^
Discharge to level II and below hospitals③		91(5.8)	2.16 ± 1.25		

Abbreviations: SD, standard deviation.

^a^*t* test was used for two-group comparisons (*t* value), and one-way ANOVA was used for multiple comparisons (*F* value).

^b^The least significant difference test was used for pairwise comparisons among three or more groups.

^c^Medical insurance includes NRCMS, UEBMI and URBMI.

*Indicates a coefficient significant at the *p *≤ .05 level of confidence.

**Indicates a coefficient significant at the *p *≤ .01 level of confidence.

Multiple linear regression analysis revealed that viewing personalized video-based health education was associated with visual and auditory impairment (*β* = −0.462, 95% confidence interval: −0.661 to −0.196), hospital length (*β* = −0.377, 95% confidence interval: −0.565 to −0.189), aphasia (*β* = −0.177, 95% confidence interval: −0.361 to 0.007), and insurance status (*β* = 0.109, 95% confidence interval:-0.103 to 0.453). The linear regression model had an *F* statistic of 20.970 (*P* < .001) and an *R*^2^ = 0.452 (Table 4).

**Table 4:** Results of multiple linear regression with personalized video-based health education as the dependent variable.

Variable^a^	Unstandardized coefficients		Standardized coefficients	*t*	*P*	95% confidence interval for *β*	
	*B*	*SE*	*β*			Lower bound	Upper bound
Constant	5.339	0.452		11.802	<0.001^**^	4.449	6.229
Visual and auditory impairment	-1.257	0.135	-0.432	-9.290	<0.001^**^	-1.523	-0.991
Hospitalization length	-0.897	0.102	-0.383	-8.799	<0.001^**^	-1.097	-0.696
Aphasia	-0.451	0.115	-0.199	-3.913	<0.001^**^	-0.678	-0.224
Insurance status							
Self-pay (Reference)	-	-	-				
Medical insurance	0.287	0.108	0.109	2.669	0.008^**^	0.075	0.499
Free medical service	0.265	0.110	0.100	2.411	0.016^*^	0.409	0.481

^a^*R*^2^ =0.453, *F*=20.970

*Indicates a coefficient significant at the *p *≤ .05 level of confidence.

** Indicates a coefficient significant at the *p *≤ .01 level of confidence.

## 4. Discussion

The above results showed that in patients with ischemic stroke, the personalized video-based health education viewing frequency is low. At the same time, the data also showed that visual and hearing impairment, length of hospital stay, language impairment, and medical insurance were found to be independent factors influencing personalized video-based health education for patients with ischemic stroke. One study [21] reported that medical workers can use information technology platforms for health education, which can improve the quality of health education by creating high-quality content and guiding patients to reliable sources.

The results showed that the per capita viewing frequency of health education videos increased during the study period, and the highest per capita viewing frequency reached 4.72 times/day. The current findings revealed that the level of knowledge and prevention practices for stroke were inadequate [22], most of the patients did not have medical professional knowledge, and it was difficult to understand the disease-related content through writing or nurses’ dictation in a short time. Effective health education can significantly improve the health knowledge and health behavior of stroke patients [23]. One study [24] reported that increased patient awareness of healthy lifestyle habits and increased interaction time between health workers and patients contributed significantly to patient capacity building for self-care. This study addresses the common habit among the population of obtaining information online in the Internet era by providing professional medical knowledge through videos and explanations with pictures and illustrations, which increases the interest in and understandability of health education [25,26]. Therefore, patients can correctly understand and master the knowledge of disease rehabilitation in a short time. In addition, the video can be watched repeatedly to strengthen the patient’s memory. According to the patient’s feedback from watching the video, the nurse can help the patient recall and understand the content of the education to improve the patient’s compliance behavior [27]. One study [28] also confirmed that video-assisted health education can be helpful in situations where nurses are understaffed. Viewing of the dietary rehabilitation education dimension, which was the most commonly viewed dimension, was associated with dysfunction in patients (71.1%). Approximately 15%–30% of stroke survivors are permanently disabled, and 20% still need institutional care 3 months after stroke onset [29]. Patients hope to recover as soon as possible, improve their quality of life, and return to society; therefore, they will take the initiative to obtain health education knowledge related to rehabilitation.

In this study, compared with patients with normal vision and hearing, patients with visual and auditory impairment watched health education videos significantly less frequently. This is because patients with visual impairment, such as blurred and double vision [30], have difficulties watching text, pictures and videos. Such patients have increased difficulty watching health education videos and cannot effectively receive video-based health education. As a result, they watch videos less frequently than people without visual impairment. Similarly, individuals with hearing impairment face more challenges accessing health-related information than those without hearing loss [31]. Due to the upper limit of the volume of the visual equipment that plays the video, patients with hearing impairment cannot hear the key content of health education in the video, so the frequency of watching health education videos is not high. In the future, we can improve the experience of video-based health education for patients with hearing impairment by adding subtitles. For patients with visual impairment, health education should be delivered orally and by touch to strengthen the effect of health education.

The average length of hospital stay in this study population was approximately 12.55 days. Patients with a hospital stay ≥13 days watched health education videos significantly less frequently than patients with a hospital stay of 0–12 days. This may be because at the early stage of the disease, patients do not know much about their disease and have an urgent desire to receive health education. However, personalized health education videos are divided into 5 dimensions—185 videos—and videos are limited. After watching the videos and mastering the health education content taught by the videos, patients had no interest in watching them again. Therefore, the longer the hospital stay was, the lower the frequency of watching health education videos was. We will further refine the video-based health education promotion plan according to the patient’s condition and discharge readiness to promote more targeted video-based health education to meet the different needs of patients at different times during hospitalization.

In this study, compared with patients with normal language function, patients with aphasia watched health education videos significantly less frequently, possibly because patients with aphasia, especially sensory aphasia, are unable to understand the language of others, their cognitive function declines [32], and they cannot understand the health education content in the videos. Video-based health education cannot effectively serve such patients, and thus the viewing frequency is low. Clinical guidelines suggest that communication can be improved by providing more time in the healthcare process for people with aphasia and adapting healthcare information to the needs of these patients [33]. Nurses can explain the health education content in stages several times after patients with aphasia watch the video-based health education materials to compensate for the lack of viewing effect.

Compared with patients with medical insurance and publicly funded medical patients, self-paying patients have a lower health education video viewing frequency, possibly because patients with medical insurance or publicly funded medical care have work units, and the national policy stipulates that they need to recover and return to normal work as soon as possible; therefore, patients with medical insurance and publicly funded medical patients pay more attention to their own health and are willing to accept health education. Self-paying patients are mostly freelancers or unemployed, and the length of rehabilitation is not much different for them, so they are insufficiently concerned with proactively seeking health education. One study [34] showed that in areas with higher urbanization levels, education levels, economic development levels, medical resources and social medical insurance coverage, the demand for health education and health promotion is more obvious. In the future implementation of health education, we should pay more attention to the health education needs of self-funded patients and provide more personalized and targeted health education content to them.

The limitations of this study are as follows. First, all included subjects were single-center patients with first ischemic stroke; therefore, to include more people in future studies, we will add patients with other diseases and patients at other research institutions. Second, the previous medical history of the study population only included hypertension, diabetes, coronary heart disease, smoking history and drinking history and did not cover the entire previous medical history of stroke patients; therefore, in future studies, we will include more risk factors for stroke patients in our statistical analysis. Third, this was a nonrandomized cross-sectional study; therefore, we will conduct RCTs to evaluate the role of personalized video-based health education. Fourth, in this study, the number of views rather than the viewing duration of the personalized video-based health education materials was selected as the dependent variable. Despite these limitations, our study provides a comprehensive profile of personalized health education for patients with ischemic stroke.

## 5. Conclusion

Patients with ischemic stroke received personalized video-based health education less frequently. Visual and hearing impairment, length of stay, language impairment and medical insurance were found to be independent influencing factors for personalized video-based health education for patients with ischemic stroke. The personalized video-based health education program constructed in this study can be used to perform keyword extraction to achieve accurate and personalized video-based health education and promotion and effectively improve the participation rate of patients in health education. The next step will be to improve the port connection between the nursing risk assessment form and the patient health education system to facilitate targeted health education for patients at high risk of falls, aspiration and stress injury; prevent adverse events; and develop a better health education model.

## Abbreviations

BMI: Body mass index
NRCMS: New rural cooperative medical scheme
UEBMI: Urban employee basic medical insurance
URBMI: Urban resident basic medical insurance
CHD: Coronary heart disease
